# Editorial: The impact of physical activity on white matter during healthy aging

**DOI:** 10.3389/fnagi.2023.1140767

**Published:** 2023-02-20

**Authors:** Elizabeta B. Mukaetova-Ladinska, Yong Liu, Annalena Venneri

**Affiliations:** ^1^School of Psychology and Visual Sciences, University of Leicester, Leicester, United Kingdom; ^2^The Evington Centre, Leicestershire Partnership National Health Service Trust, Leicester, United Kingdom; ^3^School of Artificial Intelligence, Beijing University of Posts and Telecommunications, Beijing, China; ^4^Brunel University London, Uxbridge, United Kingdom; ^5^The University of Parma, Parma, Italy

**Keywords:** dementia, aging, white matter (WM), neuroprotection, physical activity

The benefits of physical activity are well-documented throughout the human lifespan. Both the current, UK Chief Medical Officer's Physical Activity Guidelines (2019) and the U. S. Department of Health Human Services (2018) highlight their protective benefits on a range of many chronic conditions, such as coronary heart disease, obesity and type 2 diabetes, mental health problems and social isolation. The guidelines recommend similar duration of physical activity for both younger and older adults. Namely, 150 min (2.5 h) of moderate intensity activity (such as a brisk walk) or 75 min of vigorous intensity activity weekly (i.e., running), along with a combination of moderate and vigorous activity, is recommended to achieve greater benefits. Similar duration of regular physical activity is also recommended by the World Health Organization with more benefits obtained when the cumulative time of physical activity is higher (300 min per week or 5 h/weekly).

How can physical activity preserve cognitive health and reduce the risk of dementia? Sedentary life, associated with poor lifestyle quality, is the usual culprit. Regular exercise can prevent, and even treat, dementia risk factors, such as hypertension (Valenzuela et al., 2021) and diabetes (*via* improving insulin resistance) (Kumar et al., 2019). Physical activity also improves mental (depression, anxiety, stress, sleep quantity and quality) and psychosocial health (social isolation and loneliness), known risk factors for dementia (Nasisi, 2020).

In addition to lowering the dementia risk factors, physical activity also impacts on brain neuroplasticity, neurogenesis and angiogenesis. Namely physical activity, *via* reducing vascular risk factors and promoting cerebral perfusion, decreases white matter damage, which is associated with (post-stroke) cognitive decline and mortality (Hung et al., 2022).

At a molecular level, exercise promotes brain derived neurotrophic factor (BDNF) release, especially in the anterior hippocampus (Sleiman et al., 2016), the region responsible for long-term encoding and retrieval of memory. The BDNF, on the other hand stimulates synaptogenesis and neurogenesis. Regular exercise also increases the growth of new blood vessels (angiogenesis) in the brain regions where neurogenesis occurs, providing the increased blood supply that supports the development of these new neurons (Van der Borght et al., 2009). In addition, neurotrophins, including BDNF and growth nerve factor, enhance the release of acetylcholine (ACh) from hippocampal neurons, and this may influence the brain synaptic plasticity and synaptogenesis (Van der Borght et al., 2009; Voss et al., 2019) while strengthening cognition. These changes appear to be accompanied by improved memory and cerebrovascular regulation (Guadagni et al., 2020), in association with an increase of BDNF in the bloodstream (Binder and Scharfman, 2004), ensuring that brain tissue consistently receives adequate blood supply to meet its needs and preserve its function. Furthermore, moderate physical activity, i.e., treadmill exercise, reduces neuroinflammation and glial cell activation, decreases amyloid β oligomers levels and improves synaptic transmission in the prefrontal cortex in transgenic (3 ×) mouse models of Alzheimer's disease, possibly related to the inhibition of GSK3β kinase activity (Mu et al., 2022). This provides direct evidence of physical activity modulating the extent of known Alzheimer's disease hallmarks, amyloid and neurofibrillary pathology, as well as synaptic loss.

The studies included in this special edition confirm the benefits of physical exercise and extend them to promoting integrity and volume of the brain white matter. Thus, irrespectively of whether these studies were performed in animal models (Chen L. et al.), younger (Song et al.) or older (Wolf et al.; Franchetti et al.) people, physical exercise led to additional improvement of cognition and wellbeing (Song et al.; Wolf et al.), alongside with increase of total brain volume (by 6% in “super aging” older adults; Kim et al.). However, the white matter integrity in distinct brain regions appears to be dependent on the extent of physical activity. Thus, higher levels of physical activity enhance white matter integrity in brain areas that have an impact on superior memory functioning, such as corpus callosum (Kim et al.), whereas Tai Chi and walking have an impact on higher fractional anisotropy values in the right uncinate fasciculus and the left external capsule, but not corpus callosum, in comparison to the sedentary adults (Chen F.-T. et al.). It is the stimulation of higher release of glutamate and acetylcholine that underlies these psychological and cognitive improvements seen during physical activity (Harasym et al.).

These studies provide further support for the neuroprotective role of physical exercise. Thus, running, in particular, increases cerebral blood flow, reduces inflammation and enhances the expression of neurotrophic factors, i.e., BDNF and insulin-like growth factor 1 (IGF-1) (Intlekofer et al., 2013), both known to promote the development and maintenance of neural circuits. The neurotrophins similarly interact with steroid hormones, i.e., estrogen, and their interaction may underlie numerous neuronal events, such as cell survival, synaptic plasticity and dendritic arborisation. The above findings are of clinical relevance since two thirds of women in menopause complain of cognitive problems, including difficulties in concentration, memory, planning and “brain fog”, with an earlier age at menopause resulting not only in a higher frequency of complaints (Hogervorst et al., 2022) but also a higher risk of developing dementia (Rahman et al., 2020). Importantly, regular exercise is associated with improved white matter microstructural integrity, regardless of the exercise type, evidence that could guide the development and application of future prevention and intervention strategies designed to address age-related cognitive impairments during late midlife (Chen F.-T. et al.). In addition, it is older adults in the 8th and 9th decades that may have greater benefits from engaging in physical activity (Franchetti et al.), a finding that may be attributable to reducing the risk for cerebrovascular disease associated with white matter lesion load.

Fractional anisotropy, an indicator of white matter integrity tends to decrease with aging and even more with dementia. However, gaining new knowledge and skills (i.e., playing an instrument, learning a new language etc.) result in increase in white matter fibers that seems to be proportional to the number of hours spent on these activities (Andrews et al., 2021). More importantly, white matter functioning improves within a few (2–4) weeks, as shown in the case of practicing meditation (Posner, 2020). These findings have a meaningful and important role in tackling the white matter lesions associated with several modifiable dementia risk factors (i.e., diabetes, hypertension, smoking, hearing loss, obesity, depression, sedentary lifestyle, social isolation, sleep disturbances). Preliminary evidence from cross-sectional studies of treated risk factors suggests that modification of these factors could slow down negative effects on white matter microstructure.

The modifiable effects of physical activity are not only restricted to physiological aging, but also continue in people with dementia, as it is shown recently in people with Alzheimer's disease, *via* regulating some of the disease biomarkers. That is, amyloid and total and phosphorylated (p181) tau protein in plasma (Yu et al., 2022). The findings of the studies in this collection collectively suggest that regular exercise is associated with improved white matter microstructural integrity, regardless of the exercise type, age, gender. This could guide the development and application of future prevention and intervention strategies designed to address age-related cognitive impairments during late midlife (Chen F.-T. et al.). Forthcoming longitudinal and randomized control trials conducted on a larger sample of both young and older cognitively intact and people with dementia are eagerly awaited to enhance prevention and therapeutic strategies to combat human physiological and pathological cognitive aging.

## Author contributions

All authors have contributed in conceptualization of the paper, writing, and editing. All authors have approved the final version of the text for submission.
